# A New Random Walk for Replica Detection in WSNs

**DOI:** 10.1371/journal.pone.0158072

**Published:** 2016-07-13

**Authors:** Mohammed Y. Aalsalem, Wazir Zada Khan, N. M. Saad, Md. Shohrab Hossain, Mohammed Atiquzzaman, Muhammad Khurram Khan

**Affiliations:** 1 Farasan Networking Research Laboratory, Faculty of CS & IS, Jazan University, Jazan, Kingdom of Saudi Arabia; 2 Electrical and Electronic Engineering Department, Universiti Teknologi PETRONAS, Bandar Seri Iskandar, Tronoh, Perak Malaysia; 3 Department of Computer Science and Engineering, Bangladesh University of Engineering and Technology, Dhaka, Bangladesh; 4 School of Computer Science, University of Oklahoma, Norman, Oklahoma, United States of America; 5 Center of Excellence in Information Assurance, King Saud University, Riyadh, Kingdom of Saudi Arabia; University of Texas at San Antonio, UNITED STATES

## Abstract

Wireless Sensor Networks (WSNs) are vulnerable to Node Replication attacks or Clone attacks. Among all the existing clone detection protocols in WSNs, RAWL shows the most promising results by employing Simple Random Walk (SRW). More recently, RAND outperforms RAWL by incorporating Network Division with SRW. Both RAND and RAWL have used SRW for random selection of witness nodes which is problematic because of frequently revisiting the previously passed nodes that leads to longer delays, high expenditures of energy with lower probability that witness nodes intersect. To circumvent this problem, we propose to employ a new kind of constrained random walk, namely Single Stage Memory Random Walk and present a distributed technique called SSRWND (Single Stage Memory Random Walk with Network Division). In SSRWND, single stage memory random walk is combined with network division aiming to decrease the communication and memory costs while keeping the detection probability higher. Through intensive simulations it is verified that SSRWND guarantees higher witness node security with moderate communication and memory overheads. SSRWND is expedient for security oriented application fields of WSNs like military and medical.

## Introduction

Wireless Sensor Network (WSN) is formed by grouping resource constrained sensor nodes that are capable of sensing and communicating and thus can be employed in a wide variety of sensing applications [1, 2], like health, traffic and environment monitoring etc. WSNs are vulnerable to many harmful attacks due to the circumstances that sensors lack tamper proof hardware and are deployed in tough, antagonistic and unattended environments. Node replication attack or clone attack is the focus of this paper in which an adversary compromises one or more sensor nodes by physically capturing the nodes (compromising secret credentials) and then creates replicas or clones of the compromised nodes, finally, secretly and deliberately deploying clones at various positions of the network. These replicas or clones can target a wide variety of applications like border security, battlefield surveillance and fire alarms to object tracking. The adversaries can launch other insider attacks like blackhole, wormhole, selective forwarding and DoS attacks etc. [3, 4] by leveraging these replicas.

One possible solution to detect these clones is to equip the sensor nodes with built in hardware that is resistant to tampering but it is not economical to provide each sensor with a tamper proof hardware. Moreover, there may be still a possibility that a smart adversary can be able to evade tamper proof hardware. Therefore, software based clone detection algorithms can be a better solution. Software based solutions for clone detection in static WSNs can be categorized into two major classes, *centralized* and *distributed*.

**Fig 1:**
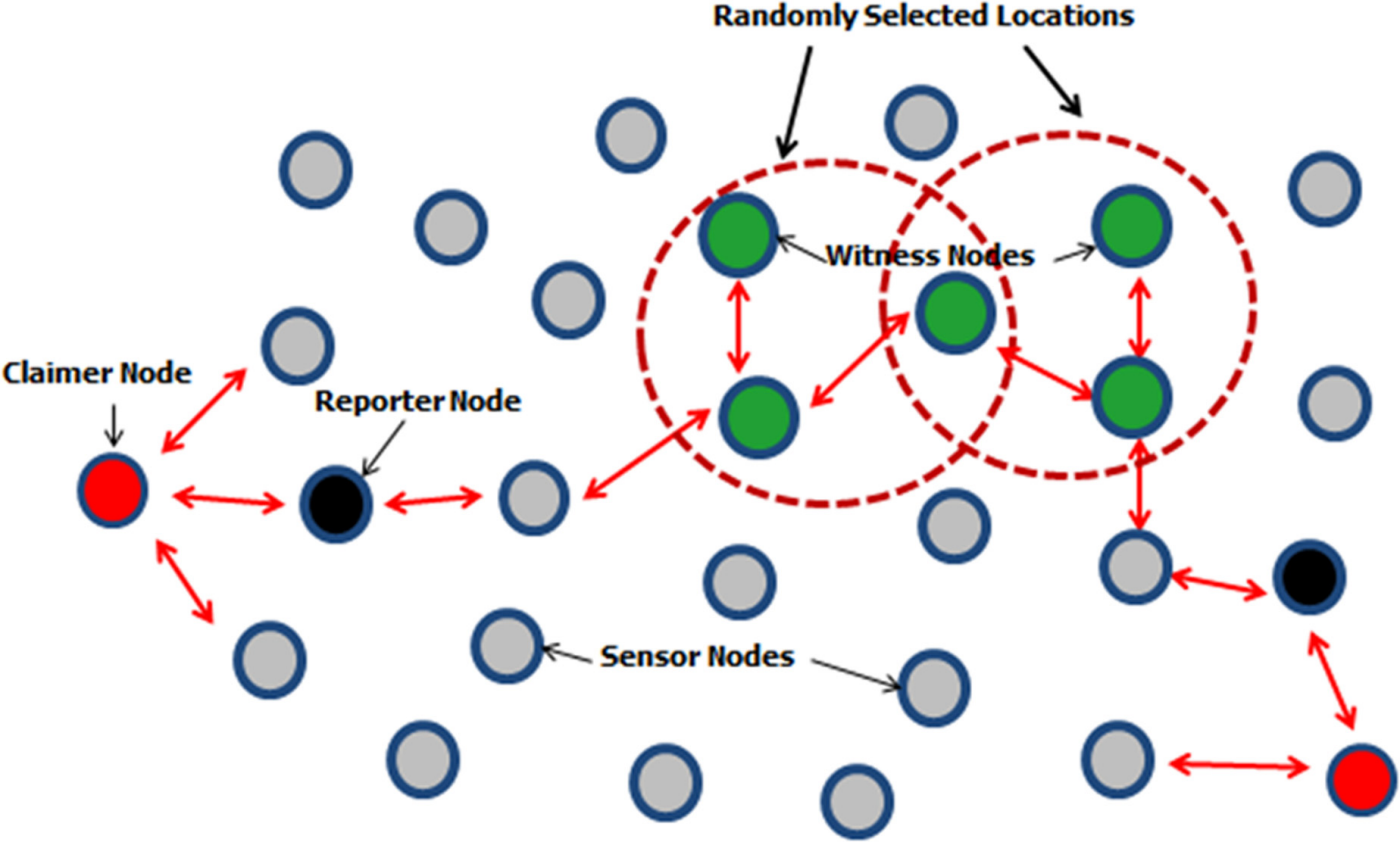
The Claimer Reporter Witness Based Framework

*Centralized* detection schemes are based Base station or cluster head, for detecting clones [5–8]. But all of these techniques have disadvantages of single point of failure and high communication costs besides achieving high clone detection rates. Thus the researchers were inclined to detect clones in a distributed manner without involving any central authority. The distributed detection schemes are called witness node based techniques [9–14] that are based on framework called claimer-reporter-witness for detecting clones. In these techniques each node (claimer node) sends its ID with locations information to its one hope neighbors (reporter node). The reporter node is responsible for mapping the claimer *id* to one or more witness nodes. The witness nodes are responsible for taking decisions for detecting clones. Witness nodes are the foundation of witness node based techniques since they are the ones capable of making decisions for identifying and uncovering the clones and therefore they are the major point of interest for adversaries. It is therefore very essential to ensure the security of witnesses. The working of witness based schemes is demonstrated in Fig 1.

There are two major concerns with the existing witness node based techniques that can undermine the security of witness nodes; *first* is the selection of witness nodes i.e. the witness nodes are selected deterministically and *second* is the distribution of witness nodes i.e. the witness nodes are distributed non-uniformly over the network. For the ideal detection of clones with ensured security of witnesses, the witness nodes should be selected smartly that an attacker won’t be able to predict about the witness nodes. Furthermore, the distribution of witness nodes should be uniform over the entire network so as to make it difficult for an adversary to guess about witnesses. Hence, there is a need to develop a technique that claims to ensure witness node security with moderate communication and memory overheads.

Encompassing the existing attempts done so far aiming to detect clones in static WSNs, RAWL [12] seems to be the most favorable approach. This is because RAWL solves the problems of other witness node based strategies by selecting witness nodes randomly and then initiating several random walks throughout the network. Besides achieving reasonable security of witnesses RAWL has still some noteworthy defects. *Firstly*, RAWL trades off costs incurred for communication and memory to achieve higher probability of detecting clones and stronger security of witnesses. *Secondly*, RAWL ensures achieving witness node intersection by initiating more random walks with longer walk steps. *Thirdly*, RAWL demands more reporters for initiating random walks that can forward the location claim to randomly selected nodes which all then initiate random walks the nodes on the passing way also become the witnesses.

RAND [13, 14] is the most recent proposal for clone detection in static WSNs which endeavors to combine simple random walk with network division, thus producing much better results than RAWL. It is verified through simulations that RAND outperforms RWAL as witness node security is ensured via dividing the network into different areas. Furthermore, this results into moderate overheads in terms of memory and communication.

Both RAND and RAWL employ a simple or pure random walk strategy [15, 16] (a.k.a., “*memory less*” or “*blind*” random walk) for selecting the critical witnesses randomly. Using simple random walk mechanism the selection of next node to be visited highly depends upon the current node and since no history or records are maintained about the visited nodes. An important reason for employing random walk is due to its simplicity and low-overhead. Also utilizing random walk for selecting witness nodes to detect clones in WSNs avoids unnecessary needs of bandwidth and energy resources which the other flooding type techniques usually consume. However, SRW has some problems. *Firstly*, previously passed nodes are revisited frequently resulting in the higher probability that the same nodes will become the witness nodes. Consequently, nodes energy will be depleted soon and then they die since same nodes are selected again and again as witness nodes. *Secondly*, frequently revisiting the nodes decrease the chances of witness node intersection, that leads to a lower probability of detecting clones as well as it makes it easy for an attacker to guess about the witness nodes since the same nodes are visited again and again.

To solve the above dilemma and thwart the noteworthy shortcomings of RAND and RAWL, we are motivated to develop a detection scheme that is more efficient in detecting clones. The contributions of this work are:

1. We propose a new kind of random walk called Single Stage Memory Random Walk and introduce a novel technique called SSRWND by combining the benefits of Single Stage Memory Random Walk with Network Division. In SSRWND when a random walk is initiated, the next node to be visited is chosen with a condition that the node should not be the node itself (current node) or the previously passed node.
2. We perform extensive simulations, comparing the results of SSRWND with RAND, RAWL and TRAWL. The simulation results show that the communication and memory costs are reduced and high security of witness nodes is ensured with increased probability of detecting clones.

The rest of the paper is organized as follows. In Section II we summarize the most related literature. In Section III we describe the assumed network and adversary models. In Section IV, we present SSRWND in detail. In Section V we present the simulation results and in Section VI we finally conclude the paper.

## Related Work

In this section, we summarize some of the most recent and most related witness node based techniques, identifying their shortcomings.

Randomized Multicast (RM) and Line-Selected Multicast (LSM) detection schemes were proposed by B.Parno et al. [9]. In RM, locations claims of claimer nodes are distributed to a set of witnesses that are selected randomly by each reporter node (one hop neighbor nodes). By exploiting Birthday Paradox [17] intersecting witness nodes are achieved that are responsible for identifying the clone detection. In LSM the nodes who forward the location claims can also server the witnesses by exploiting the network routing topology and geometric probability to find the conflicting location claims. The main problem of both RM and LSM is in probabilistic selection of the witness nodes. Moreover, LSM suffers from crowded center problem.

RED was proposed by Conti et al. [7, 8] that is comprised of two phases. The first phase is sharing a random value, *rand* among all the nodes through base station. The second phase is the clone detecting phase in which location claim is sent to a set of pseudo-randomly selected network locations. A powerful smart attacker can easily compromise the witnesses since witness node selection is deterministic. Also the infrastructure for distributing RED’s random seed may not always be available.

Single Deterministic Cell (SDC) and Parallel Multiple Probabilistic Cells (P-MPC) were proposed by Zhu et al. [10, 11]. In SDC and P-MPC each node Id is mapped to a geographical grid called cell. In SDC, the node ID is mapped to a single deterministic cell whereas in P-MPC, node ID is mapped to multiple deterministic cells (using geographic hash function) [18]. The location claims are broadcasted in each cell and the storing nodes become the witness nodes that revoke the clones form the network by identifying the conflicting claims. The selection of cell size is vital in both of these schemes since high communication costs are incurred on the selection of large cell and selecting a small cell size leads to effortless witness node compromise.

**Fig 2:**
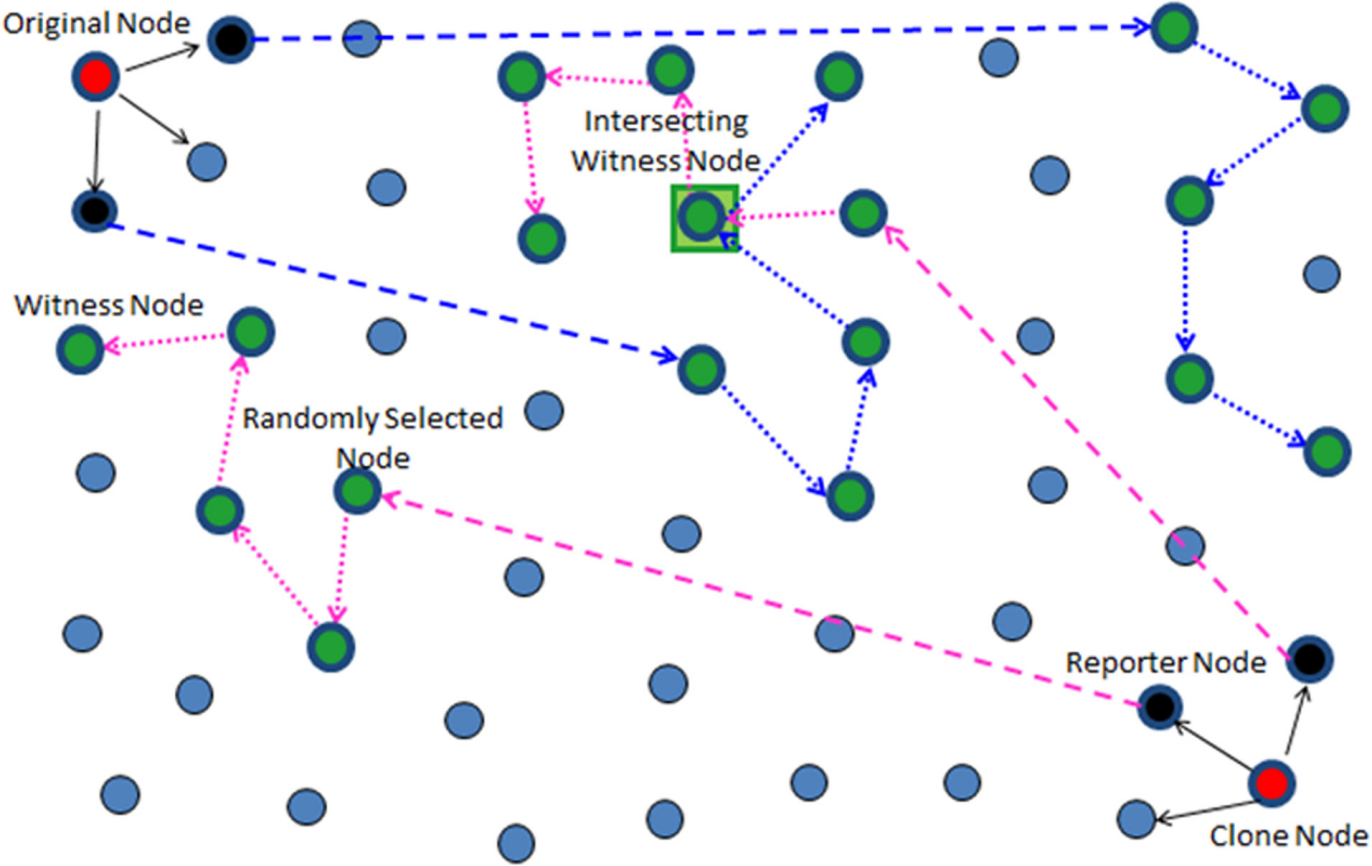
Working Principle of RAWL & TRAWL.

Random Walk (RAWL) and Table-assisted Random Walk (TRAWL) were proposed by Y. Zeng et al. [12]. In RAWL SRW is used to select witnesses which can revoke the replicated nodes form the network upon receiving the conflicting claims. TRAWL follows the same detection procedure as RAWL but memory costs are reduced by using trace table at each node. For achieving higher detection probability RAWL and TRAWL need more random walks with longer walk steps, leading to higher communication and memory costs as compared to LSM. Fig 2 demonstrates the working of RAWL and TRAWL.

**Fig 3:**
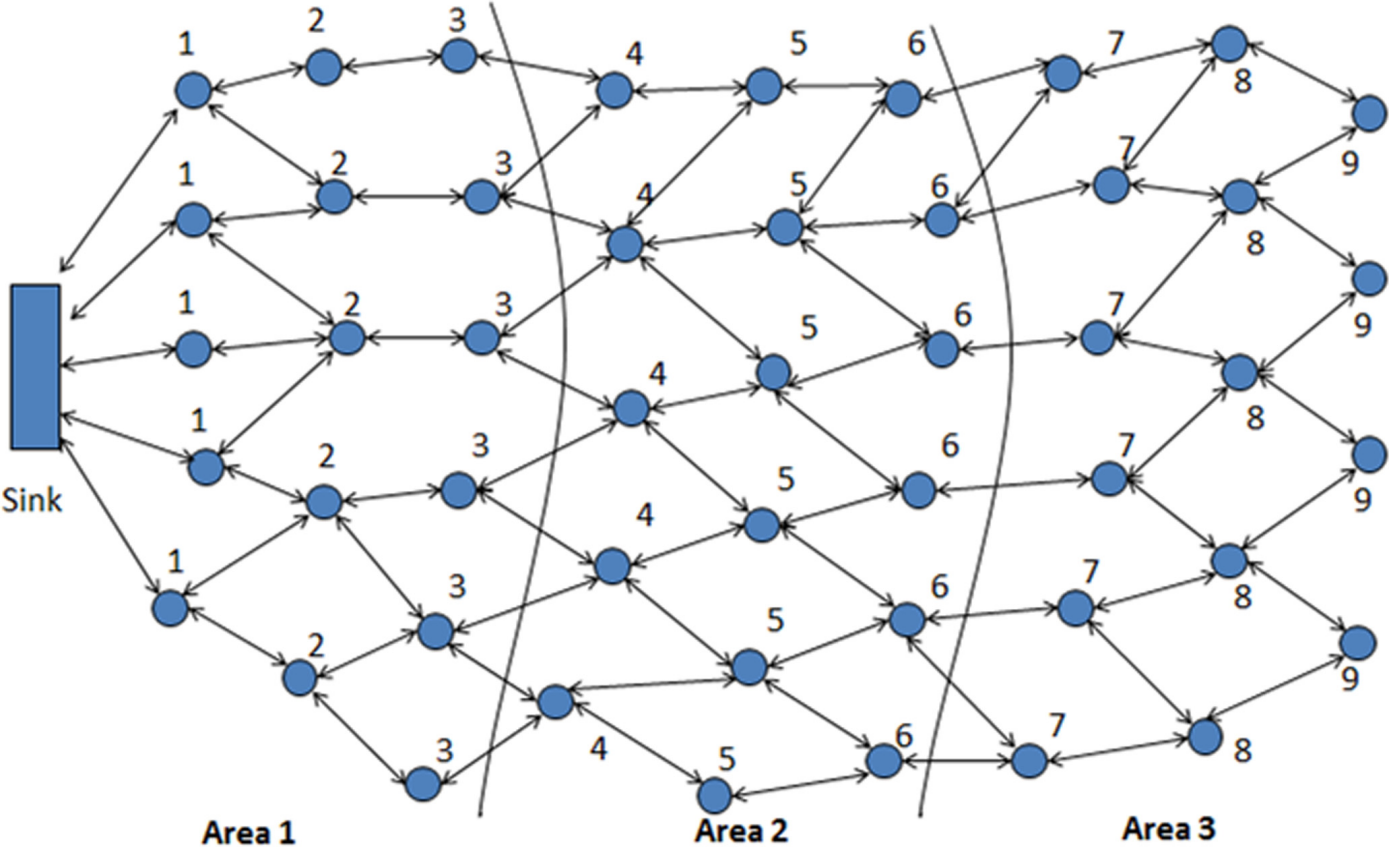
Assignment of Levels and Areas during Network Division [13, 14]

RAND [13, 14] combines SRW with network division and performs two steps, the network configuration step divides the entire network into hierarchical levels, formulating one or more levels a specific area. In the replica detection step, reporters initiate SRWs in each randomly selected area to selection of witnesses. Each pass node by random walk will become a witness node and store the location claim. The network division helps to reduce the communication and memory costs ensure the high security of witness nodes. The working of RAND can be illustrated by using Fig 3.

The contribution in [19] are reviewed and after further investigating the SSRWND protocol by theoretically analyzing the network division and selection of areas mechanism, the expected return time of random walk, security analysis and efficiency analysis are presented in this paper. The further simulations prove that SSRWND outperforms the previous schemes in-terms of high security of witnesses and detection probability with moderate overheads. Details of other more recent replica detection and authentication schemes can be found in [20–36].

## Requirements for Claimer-Reporter-Witness Node Based Schemes

Claimer Reporter Witness (CRW) based (or witness node based) Schemes are considered to be the most efficient techniques so far. However, they also have several limitations. They lack some vital requirements which should be taken into account while designing distributed witness node based techniques. In CRW-based schemes, witness nodes (intersecting witnesses) are an important element as these witnesses detect and revoke the clones. Thus, the basic requirements for designing CRW-based techniques are about the random selection, security and uniform distribution of these witnesses.

In this section, we describe these essential requirements for distributed claimer reporter witness node based schemes which should be fulfilled to make clone detection more effective and robust.

1. ***Witness Selection*:** Most importantly witnesses should be selected non-deterministically with equal probability of being witnesses. With deterministic witness selection, smart attack can be launched by an attacker who greedily chooses witnesses.
2. ***Witness Distribution*:** The second requirement is the distribution of witness nodes. The witness nodes should be distributed uniformly throughout the network
3. ***Witness Security*:** The security of witnesses can be easily compromised in deterministic schemes due to small number of witnesses and can be under the control of an attacker during the lifetime of the network. When witnesses are selected non-deterministically an attackers is unable to judge about the critical witnesses. The security of witnesses can be safeguarded by making the scheme both ID and area oblivious (No information of ID and Location) and by giving each node an equal probability of being witnesses regardless of its geographic location respectively. The detection probability of clones can be higher by certifying the security of critical witnesses.
4. ***Overheads*:** Designing protocols with lower overhead is challenging dueto the resource constrains. Energy drain of the nodes will affect the functionality the whole and if only few nodes experience high memory demand, then these nodes will start dropping packet as result of memory overflow. Developing protocols with moderate overheads and higher detection probability is very important.

## Network and Adversary Model

A large number of uniformly distributed static low cost sensor nodes knowing their location are assumed. Each node knows its geographic location by using localization schemes. Nodes are stationary each having a unique ID with a pair of identity based public and private keys and remains static until end of each protocol execution. Attackers cannot create new IDs for their replicas as nodes are protected by pair wise keys similar to [7–9, 12–14]. New nodes can replace the old or dead nodes into the network [10, 11] that need to forward their location claim to their one hop neighbors.

Adversaries are assumed to be simple but powerful that can deploy clones (deliberately) in the network created by capturing and compromising sensors. An adversary is assumed to be capable of capturing and compromising only a limited number of nodes. SWATT [37] can be employed if unlimited sensor nodes are compromised by the adversary.

## SSRWND

To describe the proposed distributed protocol SSRWND we resort claimer-reporter-witness framework. SSRWND works in the same manner as [13, 14] with the difference of employing a new kind of random walk termed as single stage memory random walk for the selection of witnesses. SSRWND combines network division with this new kind of random walk which in result not only achieves high detection probability but the overheads are also reduced as compared to RAND and RAWL. We identify that by employing Simple Random Walk in RAND, the selection of the next node at random leads to frequent revisiting of nodes that results in long delays and higher energy consumption. To avoid this problem, we propose SSRWND which selects unvisited neighbors and thus accelerates the detection process.

SSRWND performs two steps; network division and replica/clone detection. Network division starts by the tagging process in which different hierarchical levels are formed by entire network division, formulating a specific area with one or more levels. With respect to a particular sink, levels are then assigned to all the nodes, each node belonging to a certain level and area. Distance to the assigned sink constitutes a level and according to the sink configuration, different number of levels comprises each area. The network division (in levels & areas) takes inspiration from [38] where the detailed process is described. During the network division the levels and areas are assigned to nodes as shown in Fig 3.

In the beginning of replica/clone detection a signed location claim $\left\langle ID_{a},loc_{a},Sig{\left\{H(ID_{a}\|loc_{a})\right\}}_{K_{a}^{Pvt}}\right\rangle$ is broadcasted by each node (claimer node) to its neighbors, where || indicating the concatenation operation and *loc_a_* the location information of node *a*. The verification of the signature along with the plausible location of a claimer is done by each reporter upon receiving the claim. With some probability, the neighboring nodes serve as the reporters of that claimer that only forward the location claim to randomly selected nodes.

Each reporter first randomly selects area(s) through the proposed area selection mechanism (that defines how many areas reporter should select from the total number of areas) and then forwards in those randomly selected areas the location claim to randomly selected nodes depending upon the number of areas the entire network is divided into. Using Eq (1), the reporters will randomly select areas when the network is divided into odd number of areas (> = 3). Likewise using Eq (2), the reporters will randomly select areas when the network is divided into even number of areas (> = 4).

$$A_{s}=\left(\frac{A_{t}+1}{2}\right)$$ (1)

$$A_{s}=\left(\frac{A_{t}}{2}+1\right)$$ (2)

Where *A_s_* indicates the areas to select and *A_t_* represents the entire network division into total number of areas. Using Eq (3) the total number of possible combinations can be calculated for any number of areas that are unordered and without replacement.

$$\overset{A_{t}}{\underset{A_{s}}{C}}=\frac{A_{t}!}{A_{s}!\left(A_{t}-A_{s}\right)!}$$ (3)

After the selection of number of areas, any one possible combination of areas is randomly selected by the reporters (using Eq (3)) in order to forward the location claim. From any areas of the network the reporters follow the above method for selecting any combination of areas, resulting to achieve at-least one intersecting area. The network division into odd number of areas results in at least one intersecting area whereas the network division into even number of areas results in at least two intersecting areas.

**Fig 4:**
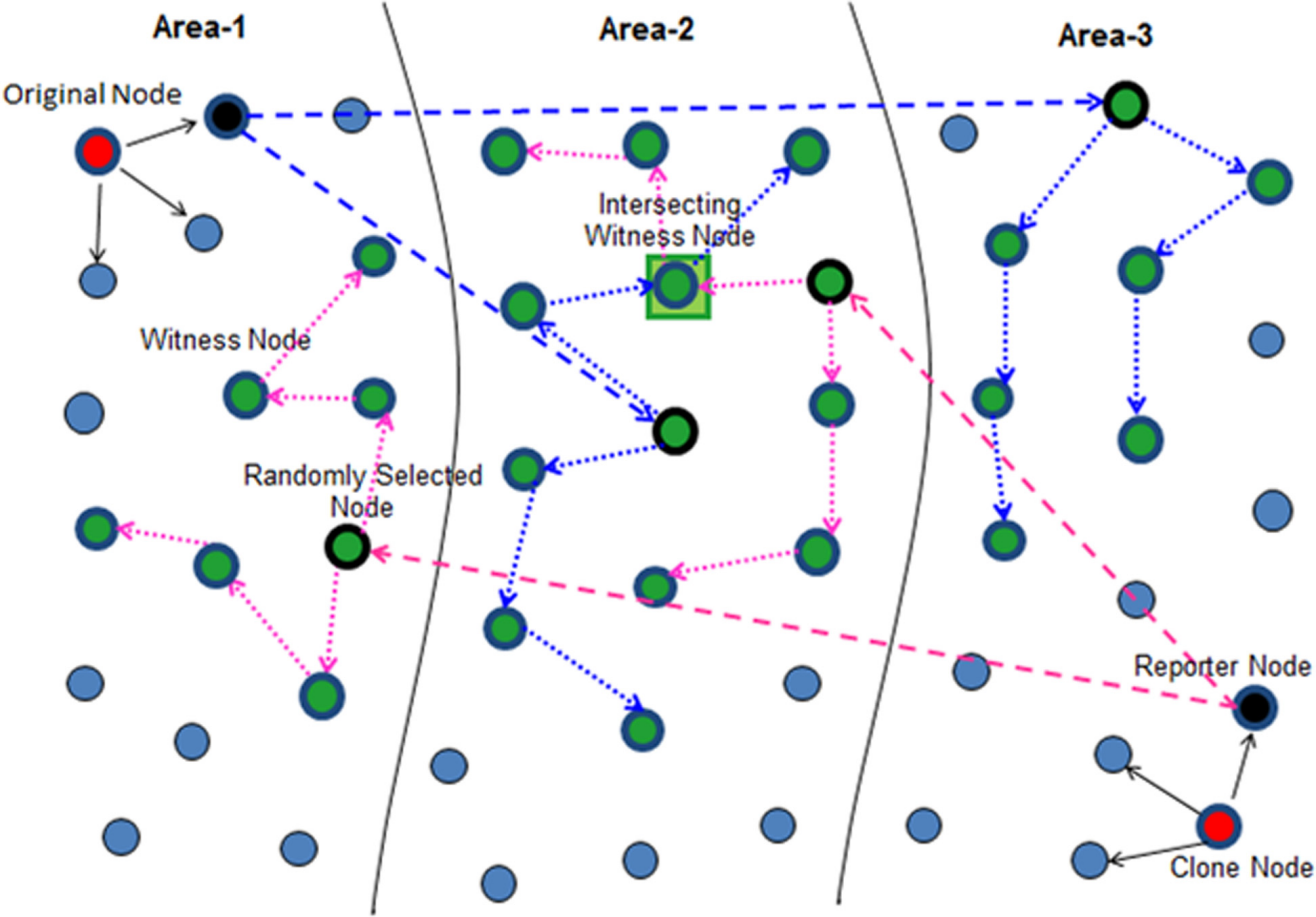
Working Principle of RAND [13, 14]

In each randomly selected area, the claim is forwarded to *g* locations by the reporter with some probability through randomly selected single node (geographic location is selected by using GPRS [39]). It is noticed in [7, 8, 12] that a random location is a better secure choice than a node *id*). Fig 4 shows the working and witness node selection of RAND.

On receiving the location claim each randomly selected node in each area verifies the signature, stores that claim and becomes the witness of that claimer. Then this first witness starts *r* single stage memory random walks of *t* steps in each randomly selected area, every passing node also becoming the witnesses. Single stage memory or conditional random walk choses the next node to be visited if the following condition is satisfied; “the node should not be the current node or previously passed node”, i.e. the location claim is forwarded to the next selected node only if at walk step *t + 1*, the node should neither be the node (previous one) at walk step *t– 1* nor the node (current one) at random walk step *t*.

**Fig 5:**
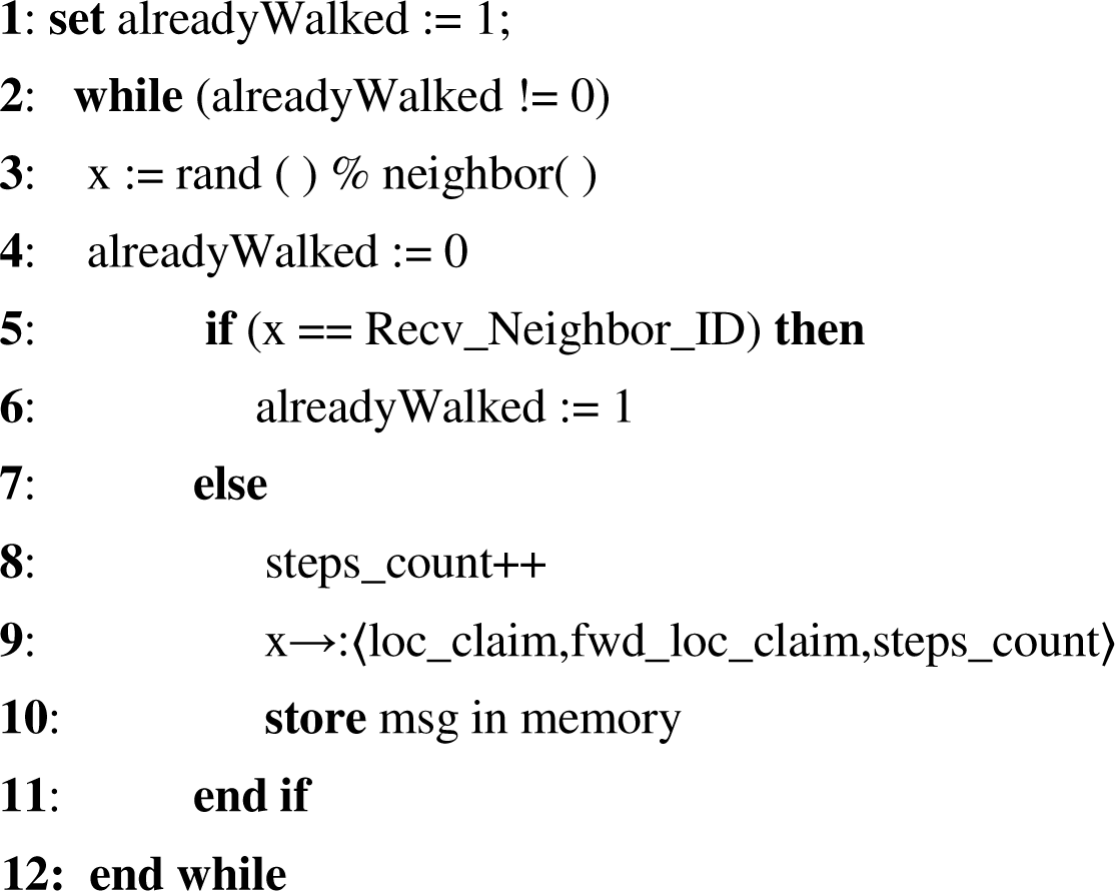
Pseudo-code for next neighbor selection in SSRWND.

The next node can be chosen by using another way which checks for the least visited node or the node that has maximum energy resources and buffer capacity (memory) [38, 40]. The least visited node is favorable in a case when the random walk reaches such a node whose all neighbors are already visited before and still the random walk steps are left. In the latter case, the network lifetime increases by evenly and uniformly utilizing the energy and buffer resources of sensor nodes. Hence the next randomly selected node is selected by each node following the above procedure and each node continues doing this until the length of random walk. On finding a conflict (two different location claims with same node ID), witness node broadcasts the two conflicting claims and revokes the replicas. Having the conflicting claims as evidence, the signatures are verified and links with replicas are terminated by every node. Fig 5 shows the pseudo code for next neighbor selection method in the proposed SSRWND protocol.

### Analysis of SSRWND

This section presents the theoretical discussion about the security and efficiency of SSRWND.

#### Analysis of Network Division and Area Selection Mechanism

Network configuration step divides the entire network into areas, assigning a particular area to each node. Supposing the network division into (A_t_) number of areas (e.g. A_1_, A_2_, A_3_, A_4_……n), and assuming the minimum number of areas to be A_t_ = 3, the network division depends upon many factors including the security of witnesses, size of the network and an area, and overall communication cost. The division of the network into minimum number of 2 areas is not feasible since through area selection mechanism both of the areas will be selected thus making it easy for an adversary to discover and identify the critical witnesses, thus, compromising the whole area with little effort for sheltering and evading detection. When the network is divided into A_t_ ≥ 3 areas, the reporters have opportunities to select different combinations of areas in more than one ways, hence predicting about reporters selection of areas (in turn about the witnesses) becomes tough for adversaries. Therefore, witnesses will be more secured with network division into minimum of A_t_ ≥ 3 areas.

Since the area size is a significant factor, it is mandatory to investigate about the network division into possible smallest and largest sized areas, so as to gain higher security of witnesses. The size of an area is also by influences the total number of nodes. We can calculate the approximate size of each area (single selected area (S_a_)) when the total number of nodes in network (N_n_) are divided by the total number of areas in the network (*A*_*t*_). It can be expressed by the formula in Eq (4).

$$\begin{array}{l} S_{a}\approx \;\left\lfloor \frac{N_{n}}{A_{t}}\right\rfloor \\ \;{where\;\{N}_{n}\geq A_{t}\;and\;A_{t}\geq 3\} \end{array}$$ (4)

The network division into three areas is shown in Fig 3. In the beginning of the detection process location claim is first sent to the neighbors by a claimer node and with some probability the location claim is then forwarded to randomly selected nodes by the neighbors that are located at different areas. The proposed area selection mechanism is used by every reporter for randomly selecting area(s) which defines that how many areas reporter should select from the total number of areas, depending upon the number of areas the entire network is divided into. With network division into odd number of areas (> = 3), the reporters will randomly select areas Using Eq (1). Likewise with network division into even number of areas (> = 4), the reporters will randomly select areas using Eq (2). Generally area selection can be performed using Eq (5).

$$Areas\;to\;Select\;(N_{sa})=\left\lfloor \frac{A_{t}+1}{2}\right\rfloor$$ (5)

The possible combinations for any number of areas are unordered and without replacement. A number of different areas (*A*_*s*_) can be selected by every reporter out of the total areas (*A*_*t*_) in a number of ways as Nc=(AtAs). Eq (3) can be used to calculate the total number of possible combinations.

The clone detection schemes based on witness nodes are highly dependent upon the way the witnesses are selected since the norms for witness selection insure the protection and security of critical witnesses through proper witness distribution, in turn increasing the detection probability of clones. Witness node based mechanisms aim to shield the witnesses so that a skilled and clever adversary remain incapable to compromise them. Aiming to achieve this much potential we propose that the network division into areas is combined with single stage memory random walk with the provision of an effective area selection mechanism to select witnesses. This concept provides uniform witness distribution in the whole network with the added security to handle smarter attackers.

#### The Expected Return Time of Random Walk

In this section different ways are explored to find out the expected return time of random walk to its already passed node (previously passed node). If there is only one random walk initiated in the network, the return time (R_i_) for random walk can be approximated as $\frac{\pi }{\log(t)},\;\mathrm{where}\;t\to \infty$, where t →∞) for infinite grid [41]. In case of finite grid and smaller random walk steps (t) the expected return time of a random walk can be calculated using following equation [42]:

$$P\{R_{i}=t\}=L_{0,0}(t)/4^{t}$$ (6)

Where *L*_0,0_(*t*) is the total number of valid paths that returns to 0 in *t* steps and *4t* is the total number of possible paths of *t* steps.

#### Security Analysis

SSRWND fulfills the optimal requirements of witness node based schemes (presented in section III). To perform the security analysis, we analyze the resiliency of SSRWND against smart attacker whose aim is to compromise the critical witness nodes (i.e., nodes that are responsible for the detection and revocation of clones). It is important to note that for non-deterministic and randomized protocols, any smart adversary needs to wait for the execution of the protocol and the clones he/she deployed in the network must become the part of the detection process by following the protocol execution.

For the additional security of witnesses, SSRWND leverages the network division into areas and in randomly selected areas initiation of random walks. Any area can be selected with equal probability by any reporter (i.e., 1/*A*_*t*_). First a random node is selected in these randomly selected areas and in each area that node will further select some random nodes for initiating (*r*) random walks, the passing nodes become the witnesses. Consequently, a skillful attacker will be impotent in finding out the critical witnesses before the protocol execution. In addition to that, the security of witnesses is guaranteed through non-deterministic and random selection of witnesses such that there will be an equal probability of each node to become a witness node without involving a base station or cluster head. As a result, it is probably very challenging for an expert adversary to guess about the witnesses.

Smarter adversaries that are aiming to locate and neutralize the crucial witnesses can be able to learn about the randomly selected areas and then in each area further determining the randomly selected starting node. In this way adversaries that are scanning and compromising all the current witness node’s neighbors can reach to the next witness and then keep on compromising the passing witnesses for ***t*** random walk steps for ***t*** times.

For a particular node, an adversary needs to compromise the total number of nodes as $O\left(\sqrt{\frac{N_{n}}{A_{t}}}\log\sqrt{\frac{N_{n}}{A_{t}}}\right),$ which an adversary is inept to do so (assuming that only a limited number of nodes can be compromised by an adversary).

Possibly an attacker is able to compromise an intermediate node of a random walk. In such a case, an attacker even cannot guess about next witnesses since he/she needs to scan all the d neighbors of the current node in order to find out the next witness node. However, an attacker can try to back-track the previously passed nodes which cannot help the attacker since each node deletes its history (previous node information) after forwarding the location claim to its next node.

#### Efficiency Analysis

To evaluate the efficiency of SSRWND, we have used three metrics, probability of detection, communication and memory cost. These metrics are chosen according to the nature of WSNs as these sensors have limited resources in terms of memory and energy. Memory and Communication costs are one of factors to be considered because they might affect life time of the WSNs as they are resource constrained. On the other hand high detection probability with moderate overheads is main objective of any detection scheme.

I **Probability of Detection:** The most important performance metric for clone detection schemes is the probability of successful detection as it is the primary security requirement of any detection scheme to detect the attack occurrence with high probability. Detection probability is defined as the total number of successful detections of clone nodes during each detection round divided by total protocol runs. The probability of replica/ clone detection is calculated by following formula.
  $$Probability\;of\;Detection=\frac{\left(Total\;\#\;of\;Successful\;Detection\right)}{\left(Total\;\#\;of\;Simulation\;Runs\right)}*100$$ (7)

The detection probability of RAWL, TRAWL, RAND and SSRWND is closely related to the number of random walk steps, since the increased number of walk steps result into higher detection probability. The reason is that: more walk steps increase the chances of intersections among the random walk steps (number of common nodes). As discussed earlier SSRWND overcomes the natural problem of simple random walk of revisiting of already passed node which in result allows the random walk to visit unpassed nodes, consequently increasing the chances of intersection.

II **Communication Cost:** The most crucial performance metric for sensor network protocols is the Communication cost since communication in WSNs uses more energy than other operations [43]. Communication cost is defined as the average number of location claim packets that are sent and received by each node during the detection round. The clone detection probability is raised by increasing the number of random walks and walk steps but correspondingly the communication costs are also increased. Comparing to RAWL, TRAWL and RAND, SSRWND requires less number of random walks and walk steps.
III **Memory Cost:** Memory cost is another important performance metric. Since low cost sensor nodes resource constrained and thus the techniques which require more storage are considered to be impractical. Memory cost is defined as the average number of location claims that are stored by each node in the areas. More walk steps mean more nods to store the location claims, SSRWND require less walk steps as compare to RAND, RAWL and TRAWL which in turn need less nodes to store the locations claims.

## Simulation Results

SSRWND, RAND, RAWL, and TRAWL are evaluated by comparing their performance in probability of clone detection, communication and memory costs incurred. For a reasonable assessment and simplify the comparison, similar simulation methodology is applied as used in [14]. In 160 x 160 square grid areas, 1024 nodes are deployed. The communication range of each node is set to 5m and each node in the network normally has degree, d = 4. In Table 1, the settings and parameters which were considered for simulations are shown.

**Table 1:** Settings and Parameters for Simulation.

Simulation Parameter	Parameter Values
Sensor Nodes	1024
Average number of neighbors	4
Deployment/ topology type	Square Grid (160m x 160m)
Communication range	5m
Location Claim size	46 bytes
Number of simulations runs	10000

For each random walk, the simulations are run for 10,000 times randomly and exclusively by dividing the network into different number of areas (e.g. 3, 4 & 5). During simulations, higher probability of detecting clones achieved by RAWL and TRAWL though initiating more long random walks is noticed. In RAND and SSRWND, the network can be divided into any number of small areas. In our experiments, the network is divided into three areas (minimum). This is because, on the division of the network into two areas, the reporters have to randomly select nodes form both the areas (according to Eq (2) of area selection), thus, the intended security cannot be achieved. However, minimum number of three areas will form an additional layer of security, making it difficult for an adversary to guess about which areas are selected for forwarding the location claim. The total communication and memory costs will increase with the division of the network into larger number of areas.

### Detection Probability

This subsection analyzes the probability of clone detection for RAWL, TRAWL, RAND and SSRWND by scrutinizing the intersecting witness nodes. Theoretically as well as through simulations we observed that at-least one intersecting area is enough for higher probability of detection and security of witness nodes and successful clone detection requires a single intersecting witness node.

**Fig 6:**
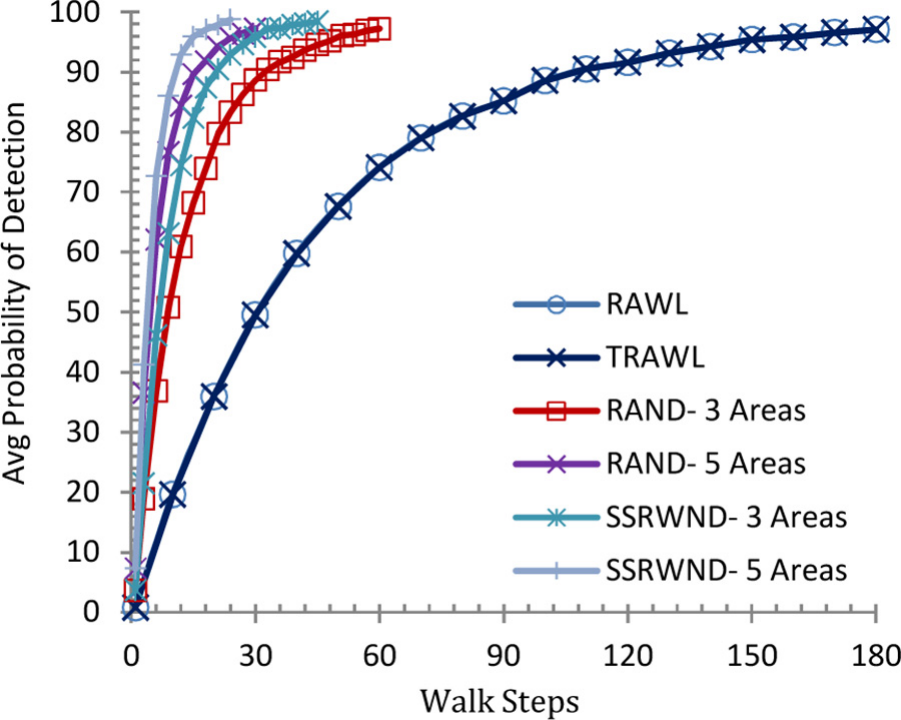
Detection Probability vs. walks step (r = 3 & # areas = 3 & 5)

**Fig 7:**
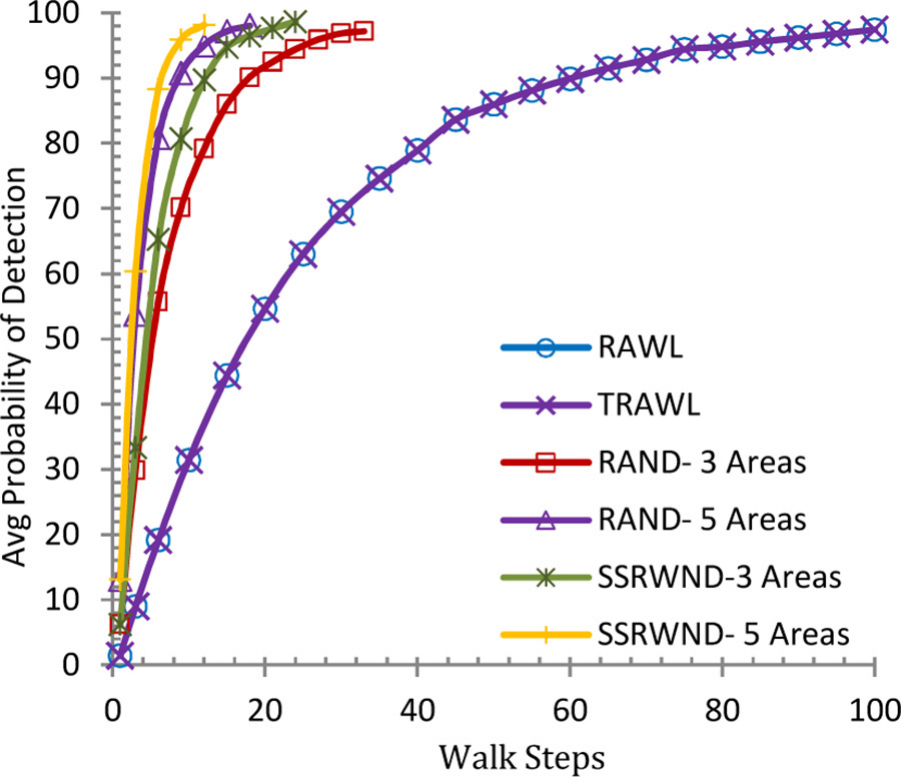
Detection Probability vs. walks step (r = 4 & # areas = 3 & 5).

It is shown in Figs 6 and 7 that the detection probability of SSRWND, RAND, RAWL and TRAWL becomes higher with the increase in walk steps incurring higher communication and memory overheads while setting the number of random walks as 3 and 4 respectively for SSRWND, RAND, RAWL and TRAWL. The network is divided into 3 and 5 areas in case of both RAND and SSRWND for the calculation of required walk steps. So, it is verified through simulations that to achieve similar detection probability, random walk steps needed for SSRWND are less in comparison to RAND, RAWL and TRAWL.

### Communication & Memory Overhead

In WSNs, communication requires more energy than other operations [43]. More number of random walks and walk steps increase the communication and memory costs. RAWL and TRAWL incur twice the communication cost of LSM so as to achieve 95% detection probability and thus trading higher communication overhead for stronger security. RAND, SSRWND, RAWL and TRAWL involve two kinds of communication costs that are incurred for detection method. Cost incurred when the location claim is forwarded to the randomly selected nodes from the reporters. And the other cost incurred is when random walks are initiated till the end of all random walk steps by randomly selected nodes. So, the total communication cost incurred is the sum of these two costs.

Communication costs of RAWL and TRAWL are of two types, *first*, when the location claim is forwarded between reporters and randomly selected nodes. And s*econd* when random walks are initiated by randomly selected nodes. In case of SSRWND and RAND, the communication costs involved are the costs incurred when a single random node is selected by the reporters in each area. The further cost incurred is when *r* random walks are initiated by each randomly selected node that is selected by the reporter in each area. When two randomly selected nodes are deployed randomly (on a unit square) the average distance between them is approximately equal to $\left(\frac{\sqrt{N_{n}}}{2}\right)$ [9].

**Fig 8:**
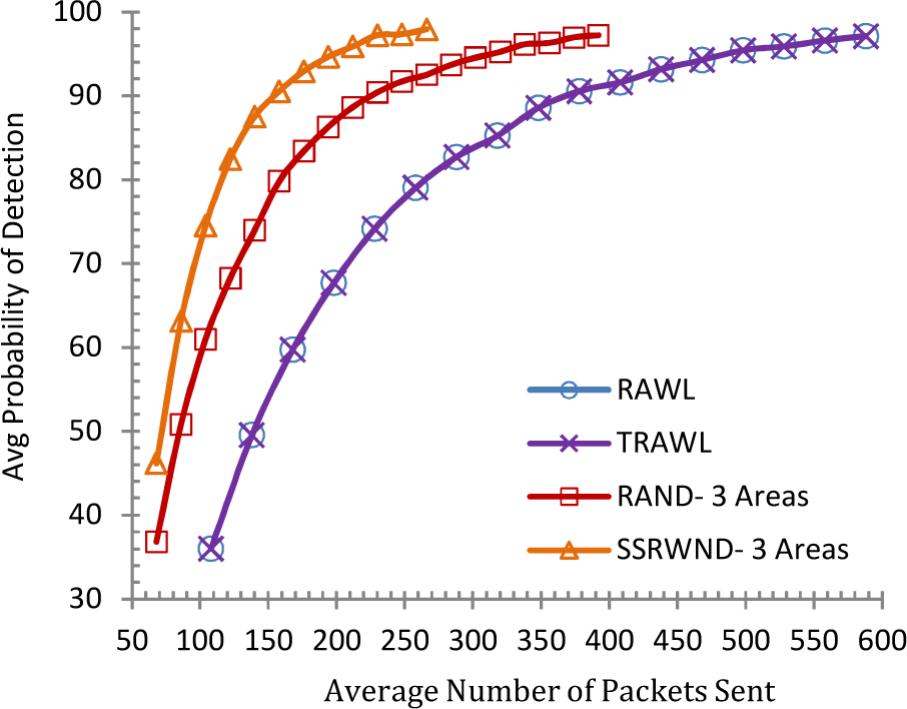
Detection Probability vs. Avg. Bytes Sent (r = 3 & # areas = 3)

**Fig 9:**
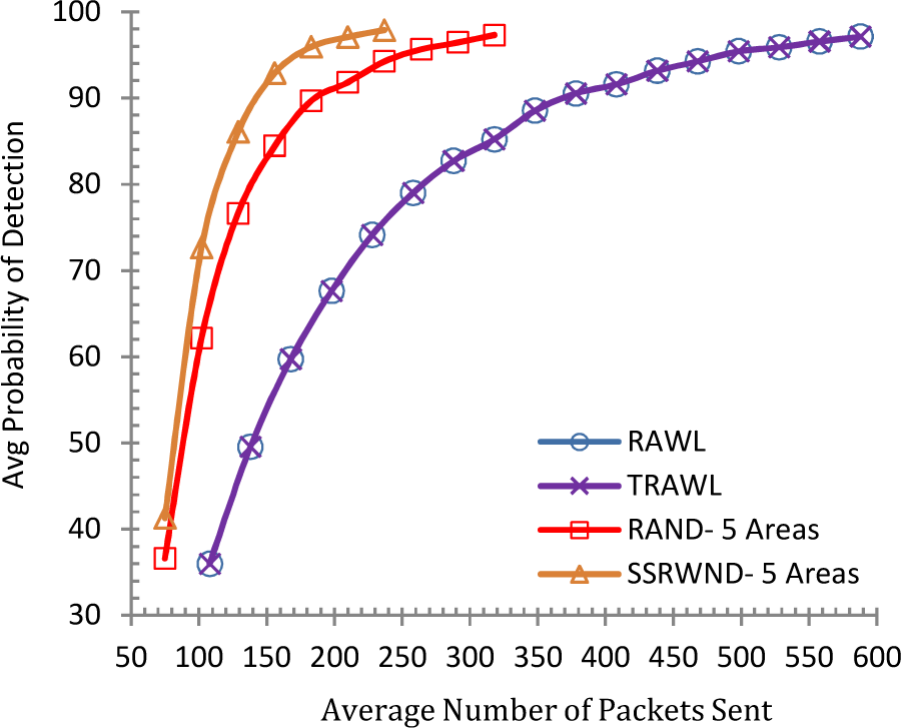
Detection Probability vs Avg Bytes Sent (r = 3 & # areas = 5)

**Fig 10:**
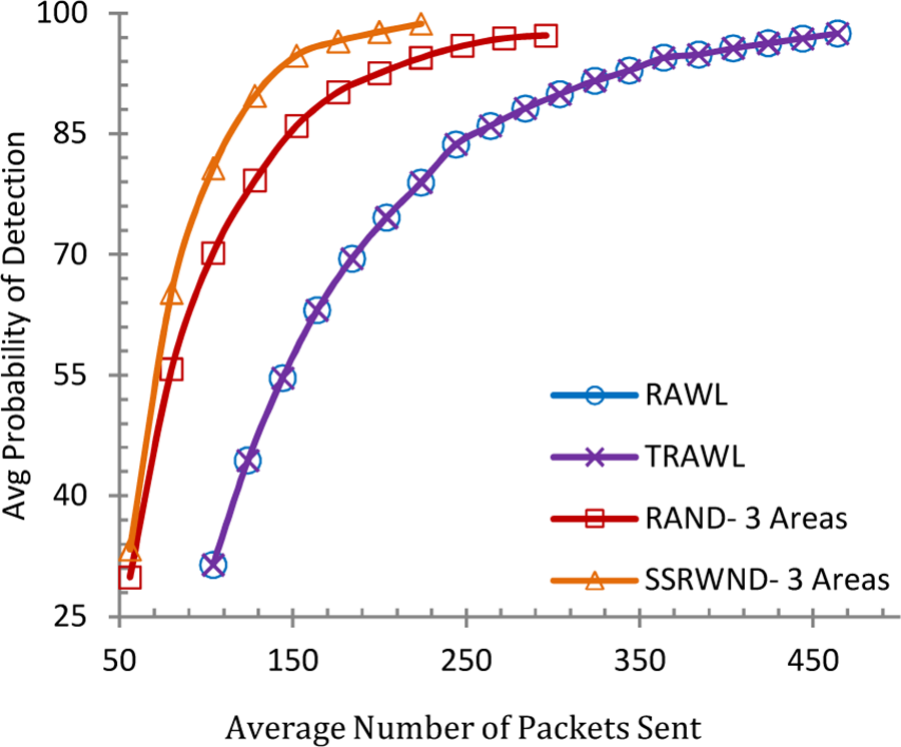
Detection Probability vs. Avg. Bytes Sent (r = 4 & # areas = 3)

**Fig 11:**
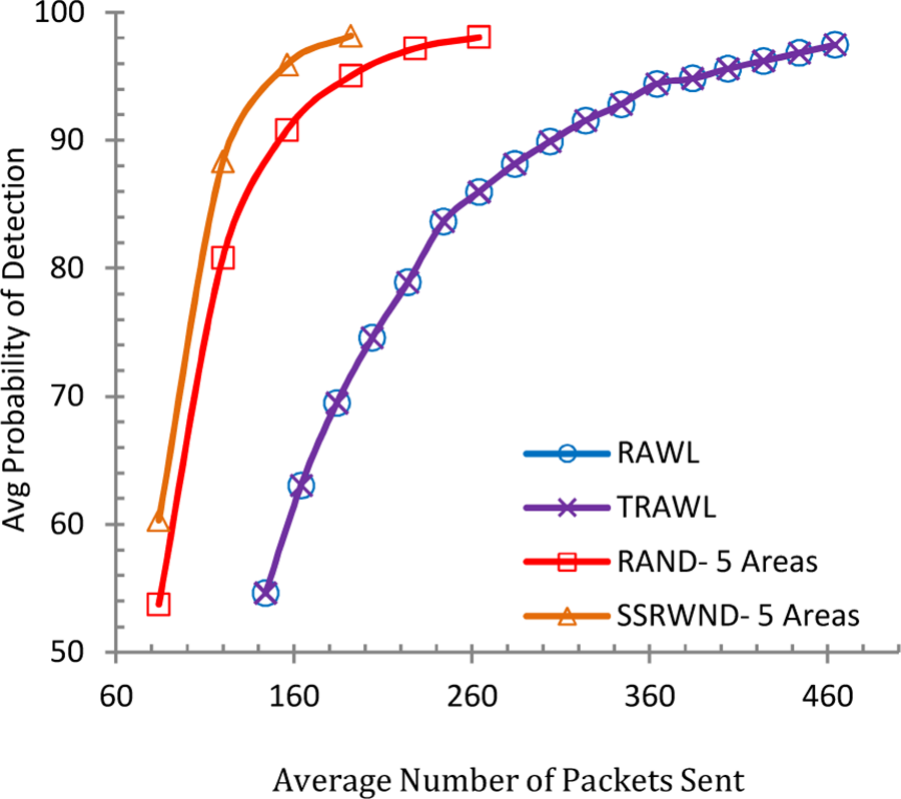
Detection Probability vs, Avg, Bytes Sent (r = 4 & # areas = 5)

Figs 8, 9, 10 and 11 show the communication overheads of SSRWND, RAND, RAWL, and TRAWL while setting the value of *r* (random walk) as 3 and 4. In case of SSRWND and RAND the number of areas is set to be 3 and 5. The results show that in order to achieve 95% detection probability SSRWND incurs lower communication costs than RAND, RAWL and TRAWL.

**Fig 12:**
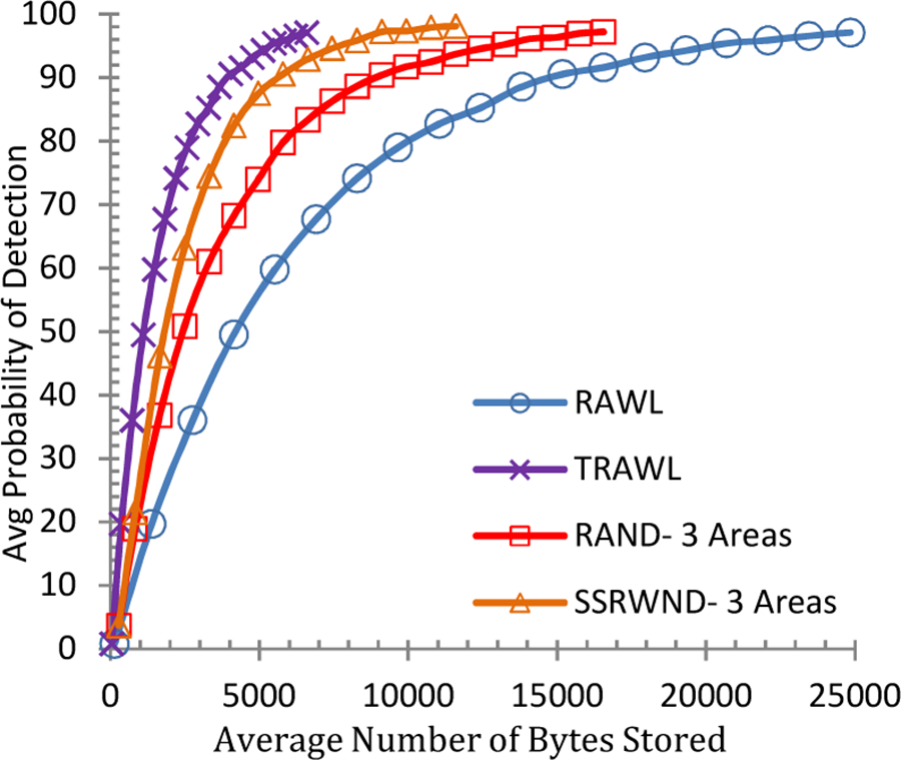
Detection Probability vs. Avg. Bytes Stored (r = 3 & # areas = 3)

**Fig 13:**
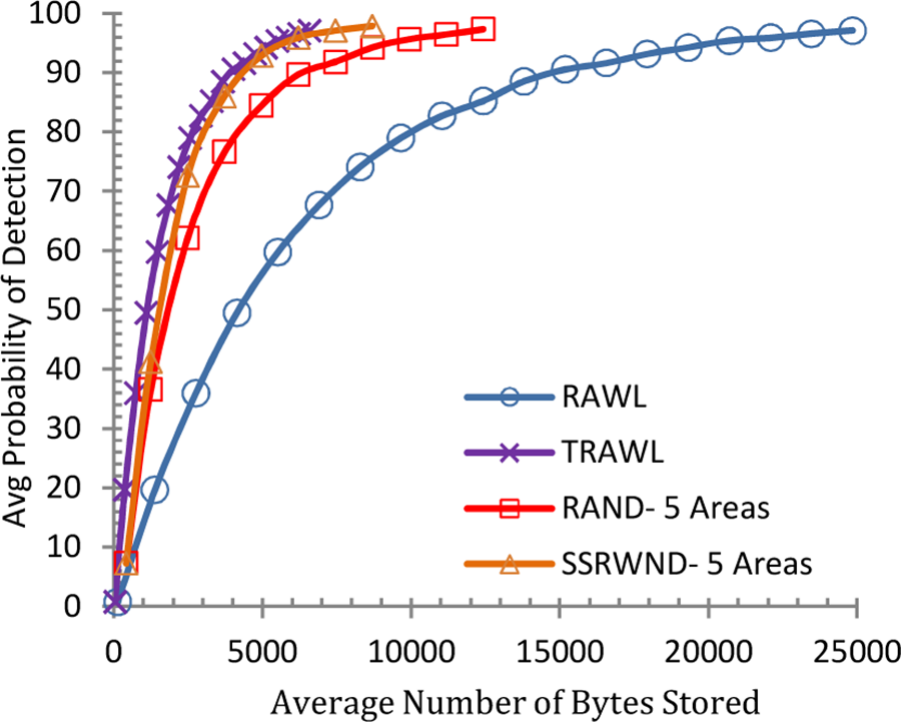
Detection Probability vs. Avg. Bytes Stored (r = 3 & # areas = 5)

Similarly Figs 12, 13. 14 and 15 show the memory overheads for SSRWND, RAND, RAWL and TRAWL. Fig 12 demonstrates that for achieving 95% detection probability, SSRWND requires 61% and 38% less memory than RAWL & RAND respectively when r = 3 and areas = 3.

**Fig 14:**
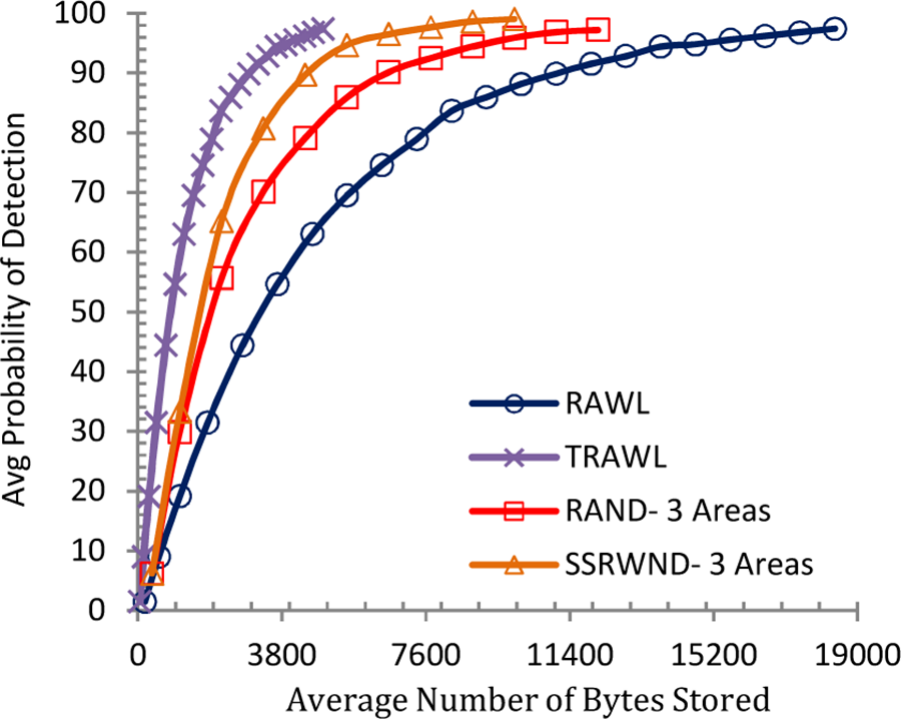
Detection Probability vs. Avg. Bytes Stored (r = 4 & # areas = 3)

**Fig 15:**
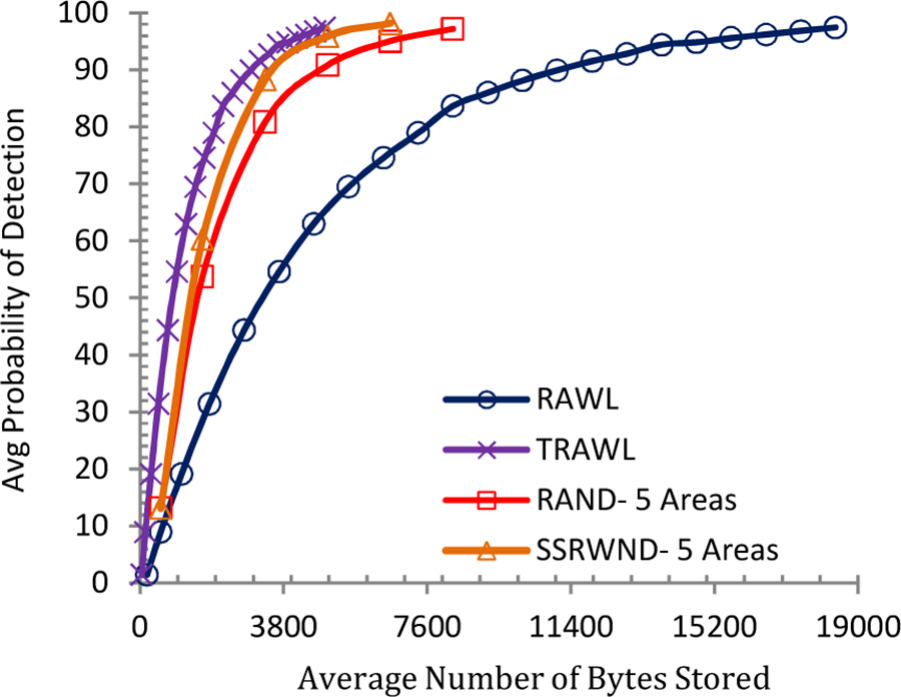
Detection Probability vs. Avg. Bytes Stored (r = 4 & # areas = 5)

Fig 13 demonstrates that SSRWND requires 72% and 39% less memory than RAWL and RAND respectively when r = 3 and areas = 5. In Fig 14 when *r* = 4 and areas = 3, SSRWND requires 62% and 35% less memory than RAWL & RAND respectively, and in Fig 15 when r = 4 and areas = 5, SSRWND requires 71% and 33% less memory then RAWL & RAND respectively. TRAWL consumes less memory than RAWL, RAND and SSRWND because it uses trace table at each node for recording the traces of random walks. The results show a minimal difference between memory overheads of SSRWND and TRAWL.

## Discussion

The communication and memory cost calculations for SSRWND, RAND, RAWL and TRAWL are shown in Table 2 in order to achieve 95% detection probability while setting *r* = 3, 4, 5 and 6 for each of 3, 4 and 5 areas in the case of SSRWND and RAND. The location claim is assumed to be 46 bytes (2 bytes for ID, 4 bytes for location and 40 bytes for signature, e.g. ECDSA [44]) for calculating the memory overhead of SSRWND, RAND, RAWL and TRAWL. The results convey that communication and memory overheads introduced by SSRWND are much lower than RAND, RAWL and TRAWL. SSRWND is also more resistant to smart and powerful adversaries than RAWL and TRAWL. In RAWL and TRAWL an adversary can be so strong that he/she can discover the whole paths of random walks by monitoring and analyzing network traffic globally. But, in case of SSRWND parallel random walks are initiated in different areas of the network which create a high level of difficulty for an adversary to find out the critical witness nodes as compared to RAWL and TRAWL.

**Table 2:** Costs incurred for Communication and Memory in achieving 95% Detection Probability.

Scheme	r	t	N_a_/N_sa_	CC-1	CC-2	Total Communication	Total Memory Cost
**RAND**	3	48	3/2	2 x 16 = 32	3 x 48 = 144	352	b/w 2.16kb & 12.94kb
	4	25	3/2	2 x 16 = 32	4 x 25 = 100	264	b/w 2.16kb & 8.98kb
	5	16	3/2	2 x 16 = 32	5 x 16 = 80	224	b/w 1.8kb & 7.19kb
	6	11	3/2	2 x 16 = 32	6 x 11 = 66	196	b/w 1.62kb & 5.93kb
	3	18	4/3	3 x 16 = 48	3 x 18 = 54	306	b/w 2.02kb & 7.28kb
	4	10	4/3	3 x 16 = 48	4 x 10 = 40	264	b/w 1.62kb & 5.39kb
	5	7	4/3	3 x 16 = 48	5 x 7 = 35	249	b/w 2.02kb & 4.72kb
	6	5	4/3	3 x 16 = 48	6 x 5 = 30	234	b/w 1.62kb & 4.04kb
	3	23	5/3	3 x 16 = 48	3 x 23 = 69	351	b/w 2.02kb & 9.3kb
	4	12	5/3	3 x 16 = 48	4 x 12 = 48	288	b/w 1.62kb & 6.47kb
	5	8	5/3	3 x 16 = 48	5 x 8 = 40	264	b/w 1.34kb & 5.39kb
	6	6	5/3	3 x 16 = 48	6 x 6 = 36	252	b/w 1.62kb & 4.85kb
**SSRWND**	3	29	3/2	2 x 16 = 32	3 x 29 = 87	238	b/w 1.62kb & 7.82kb
	4	16	3/2	2 x 16 = 32	4 x 16 = 64	192	b/w 1.44kb & 5.75kb
	5	11	3/2	2 x 16 = 32	5 x 11 = 55	174	b/w 1.8kb & 4.94kb
	6	8	3/2	2 x 16 = 32	6 x 8 = 48	160	b/w 1.62kb & 4.31kb
	3	12	4/3	3 x 16 = 48	3 x 12 = 36	252	b/w 1.62kb & 4.85kb
	4	8	4/3	3 x 16 = 48	4 x 8 = 32	240	b/w 1.62kb & 4.31kb
	5	6	4/3	3 x 16 = 48	5 x 6 = 30	234	b/w 2.02kb & 4.04kb
	6	4	4/3	3 x 16 = 48	4 x 6 = 24	216	b/w 1.62kb & 3.23kb
	3	14	5/3	3 x 16 = 48	3 x 14 = 42	270	b/w 1.62kb & 5.66kb
	4	8	5/3	3 x 16 = 48	4 x 8 = 32	240	b/w 1.62kb & 4.31kb
	5	6	5/3	3 x 16 = 48	5 x 6 = 30	234	b/w 1.35kb & 4.04kb
	6	5	5/3	3 x 16 = 48	6 x 5 = 30	234	b/w 1.62kb & 4.04kb
**RAWL**	3	150	-	3 x 16 = 48	3 x 150 = 450	498	b/w 2.56kb & 20.2kb
	4	85	-	4 x 16 = 64	4 x 85 = 340	404	b/w 2.16kb & 15.27kb
	5	51	-	5 x 16 = 80	5 x 51 = 204	335	b/w 2.92kb & 11.46kb
	6	36	-	6 x 16 = 96	6 x 36 = 216	312	b/w 2.16kb & 9.7kb
**TRAWL**	3	150	-	3 x 16 = 48	3 x 150 = 450	498	b/w 0.68 kb & 5.36kb
	4	85	-	4 x 16 = 64	4 x 85 = 340	404	b/w 0.57 kb & 4.05kb
	5	51	-	5 x 16 = 80	5 x 51 = 204	335	b/w 0.77 kb & 3.04kb
	6	36	-	6 x 16 = 96	6 x 36 = 216	312	b/w 0.57 kb & 2.57kb

r = Random walks, t= walk steps, Na = # of total areas in the network, Nsa = # of selected areas

CC-1 = Between reporter and random node (Communication cost), CC-2 = Communication cost for selecting nodes by random walks

Total Communication Cost = CC-1 + CC-2

## Conclusion

The clone detection protocols like RAND and RAWL employ SRW which revisits the already passed nodes naturally, which reduces witness node intersection and detection probability. Focusing on the problem of node revisiting, this paper presents a distributed technique called Single Stage Memory Random Walk with Network Division (SSRWND) that improves the RAND protocol by merging constrained memory random walk with network division. SSRWND achieves much better results than RAND, RAWL and TRAWL because it employs random walk with memory in which the last visited node is kept in a record so as to decrease the node revisits. SSRWND attains greater witness node security with higher probability of detecting clones and moderate overheads (communication & memory). Some of the applications of SSRWND include security oriented application fields of WSNs like military and medical etc. In future we aim to perform the scalability analysis of our proposed protocol.
